# Supplementary material for: Ultra-narrow inhomogeneous spectral distribution of telecom-wavelength vanadium centres in isotopically-enriched silicon carbide
Source: Nat Commun. 2023 Dec 19;14:8448. doi: 10.1038/s41467-023-43923-7 (PMC10730896; doi:10.1038/s41467-023-43923-7)
Supplement: Supplementary file 1 — Supplementary Information [file 41467_2023_43923_MOESM1_ESM.pdf]

Supplementary Information

Supplementary Note 1: Details of the Experimental Setup

The measurements described in the main text were performed in a home-made setup shown in Supplementary Figure 1, based on a confocal microscope.

The sample is mounted in a closed-cycle cryostat (Montana Cryostation S100). The cryostat comprises a 100x microscope objective (Olympus LCPLN100XIR, NA 0.85), tailored for near-infrared measurements, and kept at room temperature by a feedback-controlled heater. The sample is mounted on top of a stack of Attocube positioners (ANPx101). The cryostat shroud is customised to allow for an external permanent magnet to reach within 20 mm from the sample. A cylindrical (two-inch diameter, two-inch thick) Neodymium magnet, external to the cryostat (Supplementary Figure 1), is used in our experiments to generate magnetic fields up to $\sim$ 100 mT, roughly aligned along the SiC c-axis. We use a commercially available gaussmeter (Tunkia TD8620) to characterize the intensity of the magnetic field at different distances from the two-inch cylindrical magnet.

The α zero-phonon line of vanadium in SiC is optically excited by two lasers: (1) a wavelength-tunable CW telecom diode laser (Toptica DL Pro, 1270 nm-1350 nm) tuned in resonance with the zero-phonon line (ZPL) of the GS1-ES1 transition ($\sim$1278.46 nm) and (2) a green diode laser (Thorlabs PL520, 520 nm), which is used as repump laser to stabilize the V^{4+} charge state of the vanadium impurity. Both lasers are coupled into their respective excitation paths via two single-mode fibres (i.e., Thorlabs P3-980A-FC-2 and Thorlabs P3-460B-FC-2), and they are collimated by means of aspheric lenses. The excitation power and the polarization of the telecom laser are controlled by a combination of a half-wave plate and a linear polarizer (respectively, "HWP" and "LP" in Supplementary Figure 1). The frequency of the telecom laser is monitored and stabilized by means of a Fizeau-Interferometer Wavemeter (HighFinesse, WS7).

A steering mirror (Newport FSM-300-NM) followed by a 4f optical system (i.e., a telescope), composed by two plano-convex lenses, L_1 (Newport - PAC35AR.16) and L_2 (Newport - PAC13AR.16) in Supplementary Figure 1, allow the scanning of the excitation spot across the sample. In particular, the 4f telescope has the double function of expanding the collimated excitations beams (by a factor of 1.3), to fill the back aperture of the objective, while ensuring that both excitations lasers enter the back aperture of the objective through the centre, when the beams are stirred by the mirror.

The photoluminescence (PL), collected through the same excitation objective, is coupled into a single mode fibre (Thorlabs SMF28) and detected by a superconducting nanowire single-photon detector (SNSPD; Single Quantum EOS). We use a combination of iris diaphragms and two cameras, one for the telecom laser (Hamamatsu C10633, "InGaAs camera" in Supplementary Figure 1) and one for the repump laser ("Green Laser Camera" in Supplementary Figure 1), to align both the optical excitation and collection paths parallel to the c-axis of the sample.

We optically excite the V defects in resonance with the ZPL and collect only the phonon sidebands (PSB), using a glass microscope slide ("glass slip" in Supplementary Figure 1) that reflects $\sim$3% of the excitation laser and a combination of spectral filters in both the excitation and detection paths. In particular, the telecom excitation laser is spectrally filtered using two 12 nm-FWHM bandpass filters, with 1280 nm central wavelength (Knightoptical, 1280DIB25, referred to as "BP1280" in Supplementary Figure 1). An additional combination of longpass filters (i.e., Thorlabs FELH1300 and FELH0600 in Supplementary Figure 1) in the detection path, ensures the suppression of both excitation lasers, and the collection of only photons with wavelength $\geq$ 1300 nm (i.e., corresponding to the PSB of the V defects [1]).

$g^{(2)}(\tau)$ Measurements

To perform $g^{(2)}(\tau)$ autocorrelation measurements, we use a Hanbury-Brown and Twiss setup. The PL emission from the defects is split by a fiber beam splitter ("FBS", Thorlabs TW1300R5F1), with 50:50 split ratio, as indicated in the "g2 measurements" blue box in Supplementary Figure 1, and then detected by two SNSPDs. We use two channels of the TimeTagger (Swabian Instruments), to record and time-correlate the arrival times of the PL photons emitted by the V emitters. To test and characterize the photon arrival time delay of our experimental setup, we performed $g^{(2)}(\tau)$ measurements of a known single emitter source (NV centre sample).

Pulsed Measurements

To study the charge state dynamics of V defects we perform time resolved PL measurements, by pulsing both the telecom and the repump lasers (depending on the experiments, as indicated in the manuscript) and time tagging the emitted PL photons. The telecom laser is pulsed by a fibre-coupled acousto-optic modulator (AOM, Aerodiode - 1310-AOM-2), while the green laser is directly pulsed through its Thorlabs LDC205C controller. All control pulse sequences, as well as trigger signals for the time-tagger, are generated by a waveform generator (Pulse Streamer 8/2, Swabian Instruments). Finally, to time resolve the PL signal emitted by the V defects, we use a time tagger (Swabian Instruments) directly connected to the SNSPD and to the Pulse Streamer.

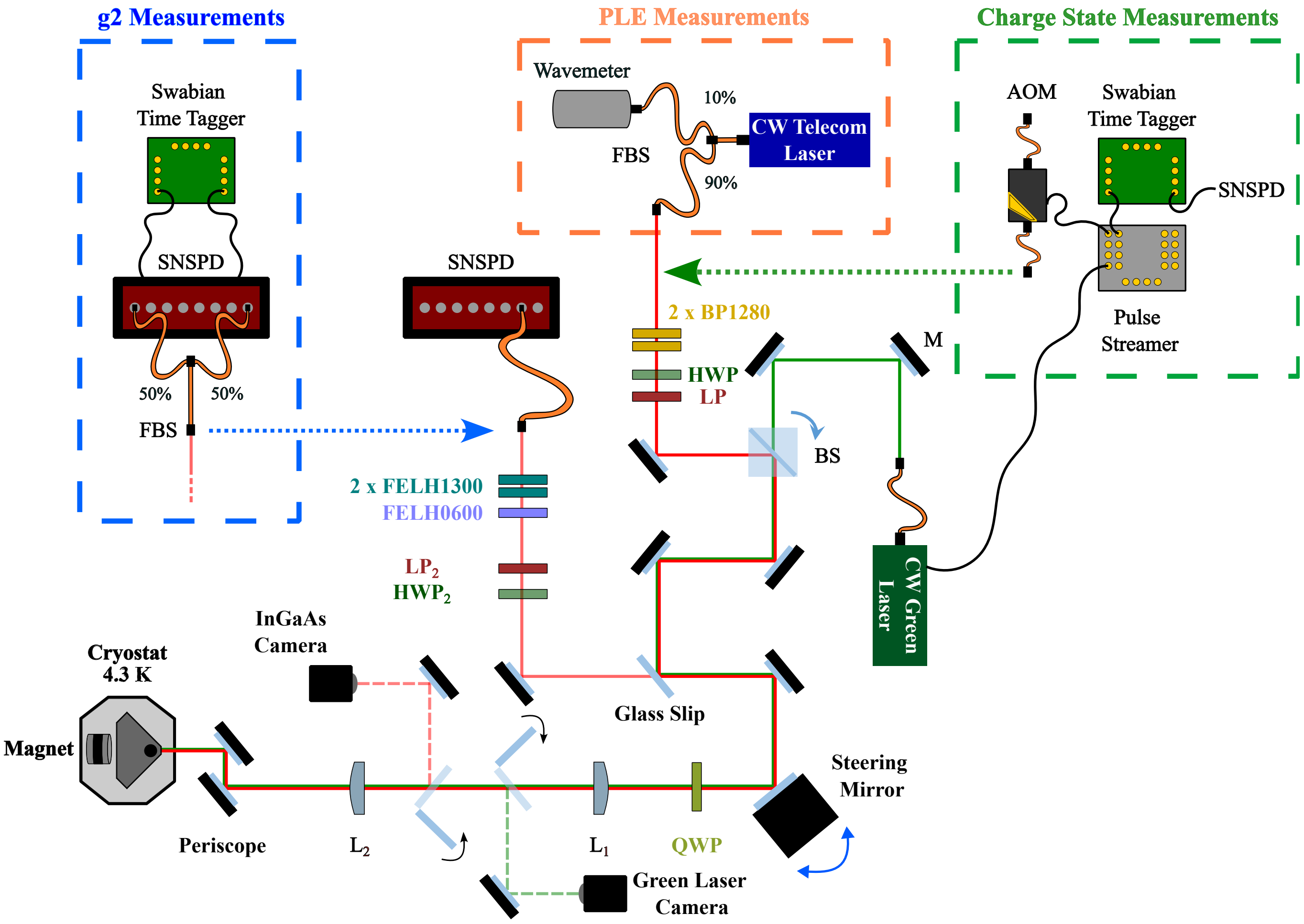

Supplementary Figure 1. **Sketch of the experimental setup.** The optical components, when not explicitly specified, are labelled as follows: **M** = Mirror, **BP1280** = Band Pass Filter, **HWP** = Half-Wave Plate, **LP** = Linear Polarizer, **BS** = Beam Splitter, **QWP** = Quarter-Wave Plate, **L1, L2** = Achromatic Doublet Lenses, **FELH0600** = Hard-Coated Long Pass Filter (Thorlabs), **FELH1300** = Hard-Coated Long Pass Filter (Thorlabs), **FBS** = Fiber Beam Splitter, **SNSPD** = Superconducting Nanowire Single-Photon Detector, **AOM** = Acousto-Optic Modulator. The small arrows close to some optical components indicate a flip mount.

Supplementary Note 2: Additional details on charge state dynamics

A. Charge transition levels

The single acceptor level (0|-) of V in 4H-SiC has been determined to be at 0.97 eV below the conduction band (CB) (E_C - 0.97 eV), by monitoring the transmutation of implanted radioactive ^{51}Cr isotope to ^{51}V using deep level transient spectroscopy [2, 3]. The energy of the V single donor level (+|0), on the other hand, has not been unequivocally assigned in the literature. Initial studies using optical admittance spectroscopy (OAS) in 6H-SiC [4] and photo-excitation electron paramagnetic resonance (photo-EPR) in both 6H- and 4H-SiC [5], assigned an energy of $\sim$ 1.6 eV above the valence band (VB) (EV + 1.6 eV) to the donor level (+|0). Later studies of V-doped semi-insulating 4H-SiC [6], found an activation energy of $\sim$ 1.6 eV from the temperature dependence of free carriers and assigned to the (+|0) donor level of V. This energy was assigned as the energy distance of the (+|0) level from the CB, E_C - 1.6 eV [6]. However, a deep donor level can compensate shallow acceptors in p-type materials but not shallow donors and, therefore, cannot pin the Fermi level in n-type materials. Thus, the activation energy measured in [6] should be related to the VB and the (+|0) level of V should be at $\sim E_V$ + 1.6 eV, in agreement with OAS [4] and photo-EPR results [5], and the Langer-Heinrich rule [7].

B. Details on dynamics of activating and deactivating V^{4+}

Most probably, the resonant laser (1278.84 nm or 0.9695 eV) does not excite electrons from the (0|-) level

of V (at E_C – 0.97 eV) to the CB. Indeed, the value of E_C – 0.97 eV for the (0|-) acceptor level of V quoted in literature [2, 3] is based on Deep Level Transient Spectroscopy (DLTS) and obtained at rather high temperature above 400 degrees, where the bandgap is at least few tens of meV (∼ 60 meV [8]) smaller than at cryogenic temperatures. Assuming that the (0|-) level is pinned to the VB one estimates that the V acceptor level is about 1.03 eV below the CB at cryogenic temperature (E_C – 1.03 eV), hence 0.97 eV optical excitation cannot ionise the V acceptor level. However, as mentioned in the main text, the 0.97 eV excitation efficiently removes electrons from the higher-lying (0|-) and (-|2-) levels of V_C (at ∼ 0.5 eV and ∼ 0.7 eV below the CB, respectively) and ionises neutral N donors. With higher power of the infrared laser more electrons are generated in the CB, which enhances the rate of capturing electrons to the acceptor level of V, leading to a higher PL quenching rate. The green re-pump laser also can ionize shallow donor and acceptor (VC) levels, but it also ionizes V^{3+} to create the bright state V^{4+}.

Thus, the decaying process of the V^{4+} PL reflects the depletion of the free-electron population in the CB created by either the green or the resonant infrared excitation. This notion is supported by PL experiments with pulsed green and infrared excitations with a delay τ between the pulses shown in Figure 4(c) in the main text. The maximum PL intensity decays exponentially with increasing delay τ because the population of the neutral V charge state decays exponentially together with the free-electron concentration.

The decay rate is sample dependent, as shown by comparison of the decay times of samples A and B (Figure 4(c) in the main text), because the free electron concentration generated by either the excitation or the re-pump is very different in these two samples, owing to different Fermi level positions, as discussed in the main text.

C. Experimental PL decay curves

In this section, we report some of the experimental PL decay curves, acquired to investigate the ionisation of the V centre in Fig 4 in the main text.

As described in the main text, we prepared the V^{4+} state with a 300 ms green laser pulse and detected the PL decay as a function of different (telecom) resonant excitation powers. Green pulses are repeated every 700 ms, a time sufficient for the detected photon intensity to be completely decayed.

In Supplementary Figure 2 (a), we report two of the decay curves acquired with the same excitation power (6 µW) on sample A (violet) and sample B (orange), respectively. In Supplementary Figure 2(b), we report PL decay curves at different excitation powers (2 µW, 8 µW and 25 µW, respectively) for sample B. To obtain the data shown in Figure 4(b) of the main text, we fitted the decay curves acquired at different excitation powers with

Supplementary Table 1. Literature parameters for the relevant KDs for the Vanadium α defect in 4H-SiC. For the ground states parameters from [9], which are based on [1] are used and for the ES parameters from [10] are used. The signs of a_k^{zz} are chosen to best describe the data. All remaining components of the g- and hyperfine-tensors are 0.

KD k	g_k^z	a_k^{zz}/h (MHz)	$a_k^{xx,yy}/h$ (MHz)	a_k^{xz}/h (MHz)
g $(1, \Gamma_4)$	1.748	232	165	0
e $(2, \Gamma_{5/6})$	2.24	213	0	75

a single exponential function, as shown in Supplementary Figure 2(b). The decay constants extracted from the fits were plotted as a function of the (telecom) resonant excitation power in Figure 4(b) of the main text. As described in the manuscript, a similar procedure allowed us to extract the decay constant shown in Figure 4(c) of the main text, as a function of the delay time (τ). Specifically, we measured the PL decays of the same V centre at different green and telecom laser pulse delays. The different delays correspond to time intervals when no illumination is present on the sample. We then fit the decays at different delays (τ) using a single exponential, and plot the peak intensity, extracted from the previous fits, as a function of the delay time (τ) (see Figure 4 (c) of the main text). For sample B in Fig. 4(c) of the main text, we repeated the experiment twice, and plotted the average of the two measurements. Error bars in Figs. 4(a) and 4(b) of the main text, correspond to the standard deviations extracted from the fits, calculated by taking the square root of the diagonal elements of the covariance matrix obtained during curve fitting.

Supplementary Note 3: Summary of the V centre electronic structure

Combining the theory developed in [9, 11, 12], we describe the structure of the optical transitions for V^{4+} using the lowest ground and excited state Kramers doublets (KDs). To this end, we model the energies of the KDs using the KD Hamiltonians

$$H_k = E^k + \frac{1}{2}\mu_B \vec{B}\mathbf{g}_k\vec{\sigma}_k + \frac{1}{2}\vec{\sigma}_k\mathbf{A}_k\vec{I} + \mu_N g_N \vec{B}\cdot\vec{I}, \quad (1)$$

where k labels the KDs, with the zero-field energies E_k of the k-th KD (in MHz), the Bohr magneton μ_B and nuclear magneton μ_N (in MHz/Gauss), the nuclear g-factor g_N, the strongly KD dependent g-tensor $\mathbf{g}_k$, as well as the hyperfine tensor $\mathbf{A}_k$, see Supplementary Table 1. Coupling to an electric field with a frequency in the vicinity of the crystal field splitting $\Delta_{\rm cr} = E^{\rm e} - E^{\rm g}$ can be described using the Hamiltonian

$$H_d^\sigma \approx \mathcal{E}(t)\epsilon \left|e,-\sigma\right\rangle\left\langle g,-\sigma\right| + \text{h.c.} \quad (2)$$

where $\sigma = \pm = \uparrow, \downarrow$ describes the pseudo-spin of the KDs as-well-as the polarization of the electric field with

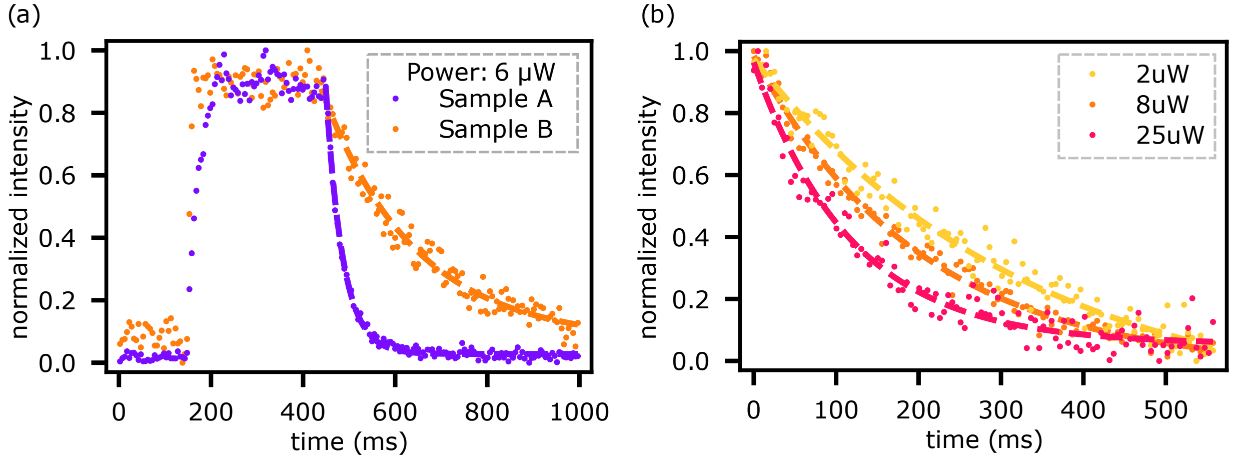

Supplementary Figure 2. Charge state measurement details. (a) Full decay curves and fittings of the V^{4+} charge state acquired under a 300 ms green laser pulse and CW telecom excitation (see inset in Figure 4(b) of the main text for details on the excitation scheme). The telecom excitation power is 6 μW for both sample A (violet) and sample B (orange). (b) Decay curves and fittings of the same PL spot in sample B at different excitation powers (see legend). Each of the curves is fit by a single exponential with a different decay constant.

amplitude $\mathcal{E}(t)$ within the rotating wave approximation. Here we only take the dominating part of the transition that conserves the spin into account and thereby neglect the much weaker spin-flipping transition possible due to the spin-orbit coupling. [The driving field should fulfil $|\epsilon\mathcal{E}| \ll \min_i |\Delta^i_{\rm so}|$ for the RWA]

To then model the spectra we assume that each of the hyperfine transitions has the same width γ. We therefore quantify the transition intensity of each of the hyperfine transitions as

$$a\frac{\gamma|\langle g,\sigma_g,m_g\|g,\sigma\rangle\langle e,\sigma\|e,\sigma_e,m_e\rangle|^2}{4(\omega-E^e_{\sigma_e,m_e}+E^g_{\sigma_g,m_g})^2+\gamma^2} \tag{3}$$

where we use a as a fit parameter independent of the hyperfine states (but depending on B). The hyperfine states are labeled according to their main spin σ_k and nuclear contribution m_k but are made up of different spin, orbital and nuclear states. Analytic expressions for the states and energies can be found in [9].

To fit the data of Figure 2 of the main text, we sum all the allowed transitions for a given polarization σ and add a global offset o (dependent on B), reusing existing hyperfine parameters [10, 12] the remaining parameters to determine the hyperfine linewidth γ, the crystal splitting Δ_{cr} (which can vary from defect to defect), as well as the magnetic field dependent fit parameters $a(B), o(B)$. Using a least squares fit weighted by the inverse Poisson error of the data, we find $\gamma = 1038 \pm 7$ MHz and central transition frequency $\Delta_{cr} = 234425594 \pm 4$ MHz.

We highlight that the narrowing of the hyperfine transitions suggests that the signs of the a^{zz}_k with $k = e, g$ are the same and that the measurement confirms the theory predicted polarization dependent selection rules.

Supplementary Table 2. Measurement setup dependent fit parameters. The unit of $a(B)$ is due to the unit of the Lorentzian being MHz.

Parameter	0 Gauss	600 Gauss	1000 Gauss
Amplitude $a(B)$ (GHz/s)	22.6±0.2	19.5±0.2	23.1±0.2
Offset $o(B)$ (1/s)	24.9±0.3	23.4±0.3	27.6±0.3

Supplementary Note 4: Additional information on the inhomogeneous spectral distribution

A. Comparison between different emitters

In Supplementary Table 3, we compare the inhomogeneous distribution and lifetime-limited linewidth for different quantum emitters in different materials platforms. Given the goal of bringing any two quantum emitters into resonance, the important figure of merit here is the ratio η between the inhomogeneous distribution of emitters and the linewidth. We take the ideal case of a lifetime-limited linewidth, computed from the optical lifetime τ as $1/(2\pi\tau)$: a linewidth as close as possible to the lifetime limit is crucial to achieve high-visibility quantum interference.

We include quantum emitters from different material platforms, including deep-level point defects in diamond, SiC, silicon and rare-earth ions in crystals. With an inhomogeneous distribution of $40-50$ GHz, the NV centre in diamond scores $\eta > 3000$, i.e. a lifetime-limited line of 13 MHz may have to be tuned across more than 3000 linewidths to bring any NV into resonance with any other NV. The silicon vacancy in diamond (SiV) and silicon carbide ($V_{\rm Si}$) achieves a better value for η, ~ 100 and > 500, respectively. It's important to stress that

SiV in diamond features first-order insensitivity to strain and electric fields due to inversion symmetry. We did not include the divacancy in SiC in the Supplementary Table, as we could not find an experimental value for its inhomogeneous broadening in the literature: given that its electronic structure and spin stare are identical to the NV centre in diamond, we however expect similar numbers. As we have seen, the inhomogeneous distribution for vanadium in SiC can be reduced from several GHz ($\eta > 10^4$) in standard material to $\sim$ 100 MHz ($\eta \sim 100$) in isotopically-enriched SiC. Recent work on the T centre in Si has demonstrated extremely narrow inhomogeneous broadening, down to 60 MHz [13], in ensemble experiments ($\eta \sim 350$): the broadening is however much larger when creating single emitters by implantation ($\eta > 5000$) [14]. Rare-earth ions show outstanding coherence of their optical transitions, with inhomogeneous broadening down to the MHz regime, in some cases (e.g. ensemble $^{153}Eu^{3+}:EuCl_3 \cdot 6H_2O$ in the Supplementary Table 3). Their long optical lifetimes, in the ms regime, with lifetime-limited linewidths in the few tens of Hz regime however results in quite high values for η.

B. Experimental determination of inhomogeneous distributions

In this section, we present additional details about the estimation of the inhomogeneous distribution for emitters in the sample, described in Figure 3 of the main text. We performed a sequence of maps of the same area at different detunings of the excitation laser (see for example Supplementary Figure 3). A custom python code automatically detects photoluminescence spots in each map and plots the integrated counts for each spot as a function of the detuning of the excitation laser (see for example Supplementary Figure 4). The photoluminescence peak for each spot is then fit with a Gaussian function. The single emitter nature of each spot was not checked, so some of the spots may correspond to multiple emitters.

We plot the sequence of maps and the corresponding spectral peaks for each spot (with the fit) for four regions (labelled A1-A4) in sample A (isotopically-enriched) and one region in sample B (natural abundance of isotopes), as follows:

- region A1: PL maps in Supplementary Figure 3 and spectra in Supplementary Figure 4.
- region A2: PL maps in Supplementary Figure 5 and spectra in Supplementary Figure 6.
- region A3: PL maps in Supplementary Figure 7 and spectra in Supplementary Figure 8.
- region A4: PL maps in Supplementary Figure 9 and spectra in Supplementary Figure 10.
- sample B: PL maps in Supplementary Figure 11, 12, 13 and spectra in Supplementary Figure 14, 15.

For the four regions in sample A, the centres of the distributions of central frequencies vary slightly between the different regions: f_{A1} = 227 ± 105 MHz, f_{A2} = −41 ± 109 MHz, f_{A3} = −25 ± 76 MHz and f_{A4} = 712 ± 97 MHz. As evidenced by the standard deviations within each region, the central frequencies are very narrowly distributed over about 100 MHz. Note that the PLE maps for region A4 were acquired after a thermal cycle of warm-up to room temperature and cool-down to 4 K, which may have affected the sample strain. For the defects observed in the area D of the sample, we confirm the ultra-narrow inhomogenous broadening by high-resolution PLE spectroscopy of six different centres (Supplementary Figure 16 b). The PLE spectroscopy measurements further corroborate the results of the automated statistical analysis.

Supplementary Table 3. Summary of inhomogeneous distribution for several spin-active quantum emitters

centre	wavelength	lifetime	Fourier linewidth	inhomogen. distr.	η
NV:diamond (single)	637 nm	12 ns	13 MHz	~40-50 GHz [15]	>3000
SiV:diamond (single)	750 nm	1.7 ns	94 MHz	~10 GHz [16, 17]	107
V_{Si}:SiC (single)	861 nm	6 ns	27 MHz	~15 GHz[18]	565
V:SiC (single)	1278 nm	167 ns	0.95 MHz	>10 GHz	>10,000
**V:^{28}Si^{12}C** (single)	**1278 nm**	**167 ns**	**0.95 MHz**	**~100 MHz**	**105**
Er^{3+}:Si (single)	1536 nm	~ 0.186 ms [19]	~ 856 Hz	~0.5 GHz [19]	$\sim 0.6 \cdot 10^6$
Er^{3+}:YSO (single)	1550 nm	~ 11 ms [20]	~ 14.5 Hz	~20 GHz [21]	$> 1 \cdot 10^9$
T centre:Si (single)	1326 nm	940 ns [13]	169 kHz	~1 GHz [14]	5900
G centre:Si (single)	1280 nm	~ 36 ns [22]	4.4 MHz	>100 GHz [23]	>27000
T centre:^{28}Si (ensemble)	1326 nm	940 ns [13]	169 kHz	60 MHz [13]	355
G centre:^{28}Si (ensemble)	1280 nm	~ 36ns [22]	4.4 MHz	72 MHz [24]	16
$^{171}Yb^{3+}$:Y_2SiO_5 (ensemble)	~ 980 nm	~ 1ms [25]	~ 160 Hz	~ GHz [25]	$> 6 \cdot 10^6$
$^{153}Eu^{3+}$:$EuCl_3 \cdot 6H_2O$ (ensemble)	579.7 nm	~ 2ms [26]	~ 80 Hz	25 MHz [27]	$> 3 \cdot 10^5$
Eu^{3+} molecular crystal	580.38 nm	~ 540 µs [28]	~ 295 Hz	~ 200 MHz [28]	$> 6 \cdot 10^5$

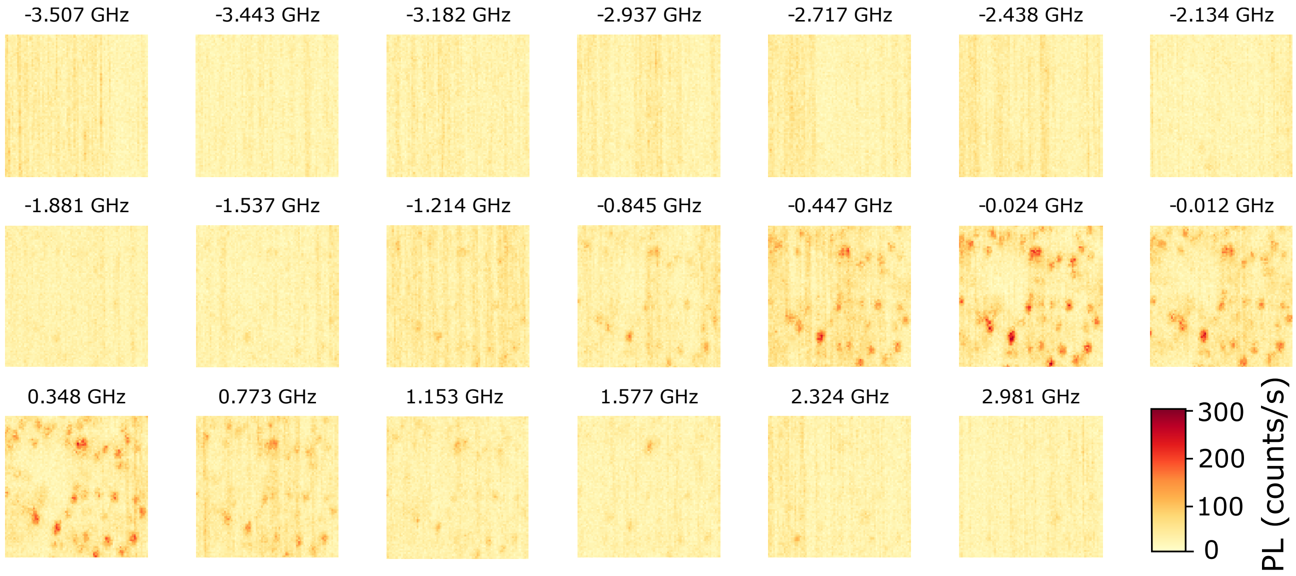

Supplementary Figure 3. **PLE maps (region A1, sample A).** Photoluminescence maps for different detunings of the telecom excitation laser for the isotopically-enriched sample. Each map shows a 12 µm by 12 µm area. For these measurements, we use an integration time of 1 s per step, an excitation power of 14 µW for the repump laser, and 4 µW for the telecom laser.

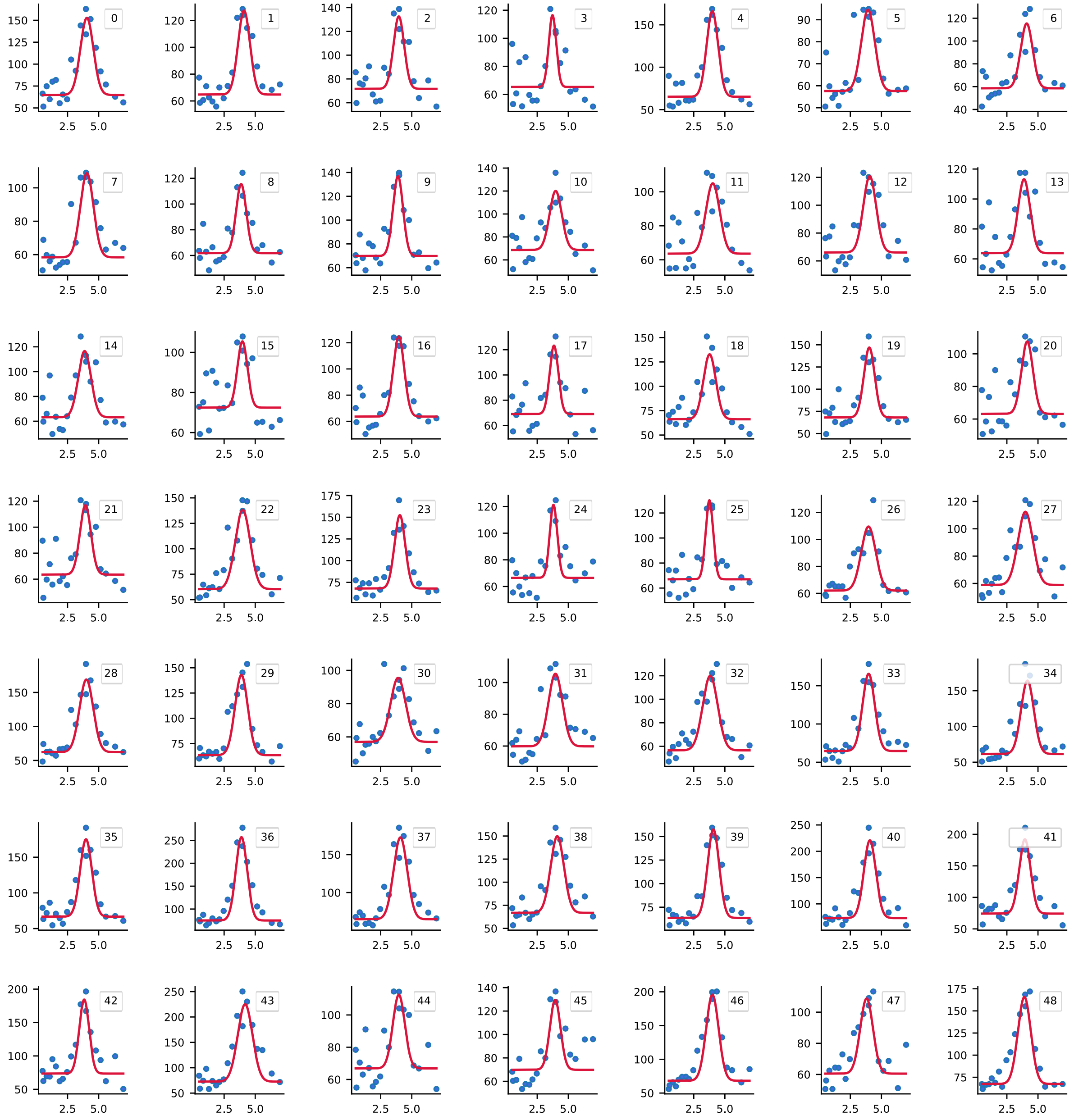

Supplementary Figure 4. **PLE spectra for each spot (region A1, sample A)**. Fitted spectra from 48 spots in Supplementary Figure 3. For each sub-plot, the x-axis shows the excitation frequency in GHz, and the y-axis the number of detected counts per second.

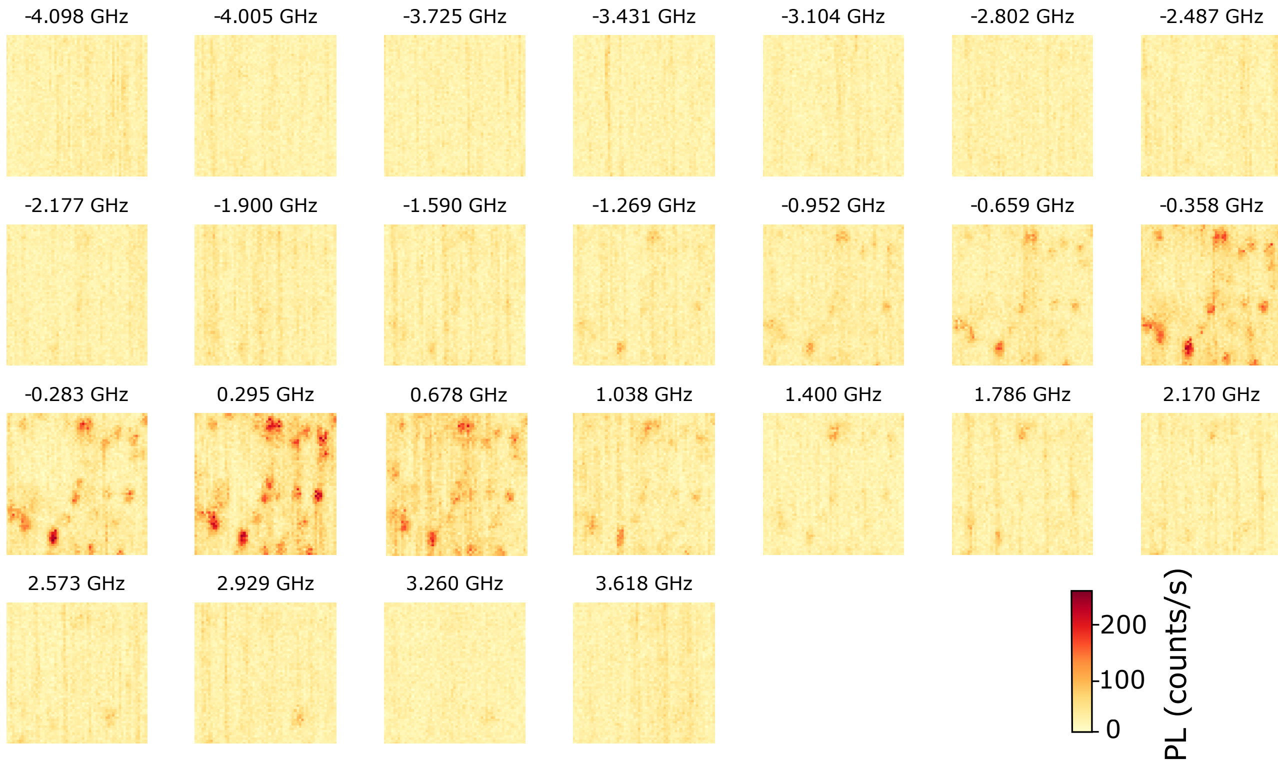

Supplementary Figure 5. **PLE maps (region A2, sample A).** Photoluminescence maps for different detunings of the excitation laser for the isotopically-enriched sample. Each map shows a 10 µm by 10 µm area. For these measurements, we use an integration time of 1 s per step, an excitation power of 14 µW for the repump laser, and 4 µW for the telecom laser.

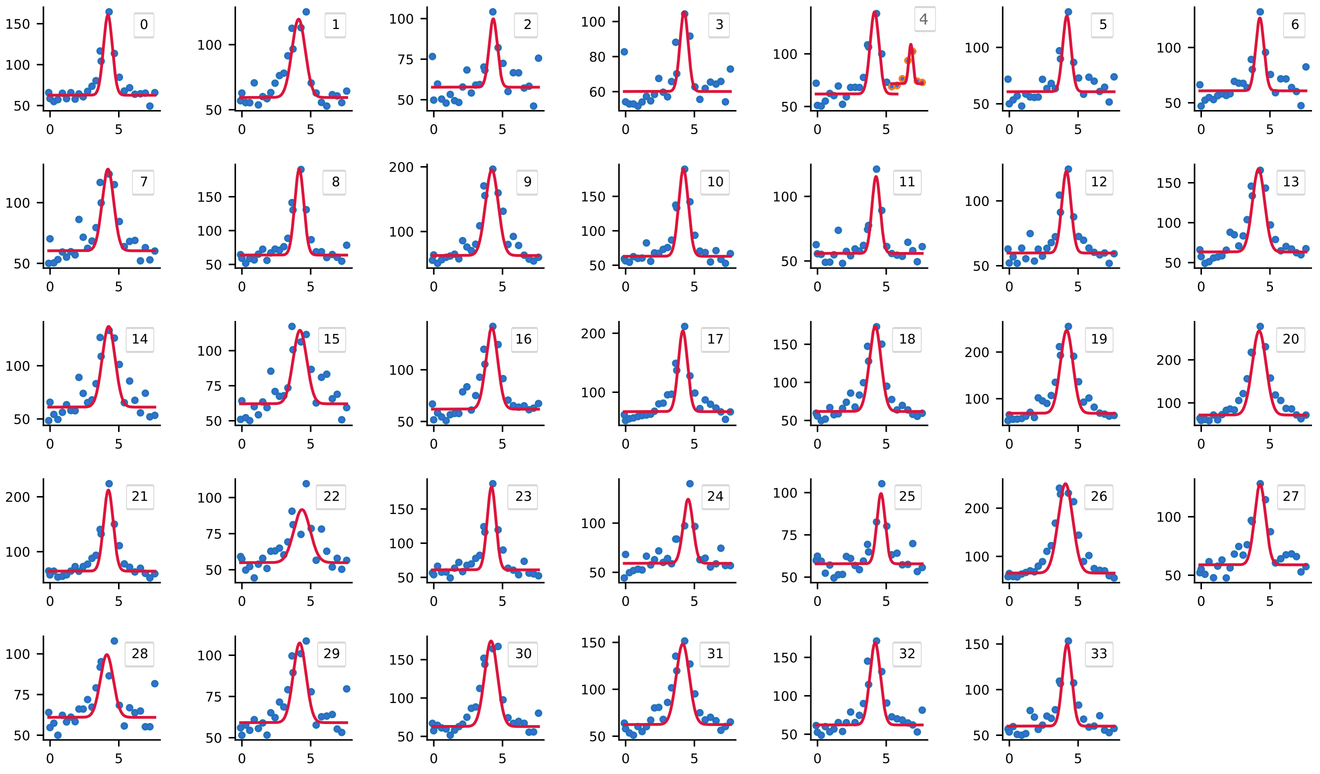

Supplementary Figure 6. **PLE spectra for each spot (region A2, sample A)**. Fitted spectra from 33 spots in Supplementary Figure 5. For each sub-plot, the x-axis shows the excitation frequency in GHz, and the y-axis the number of detected counts per second.

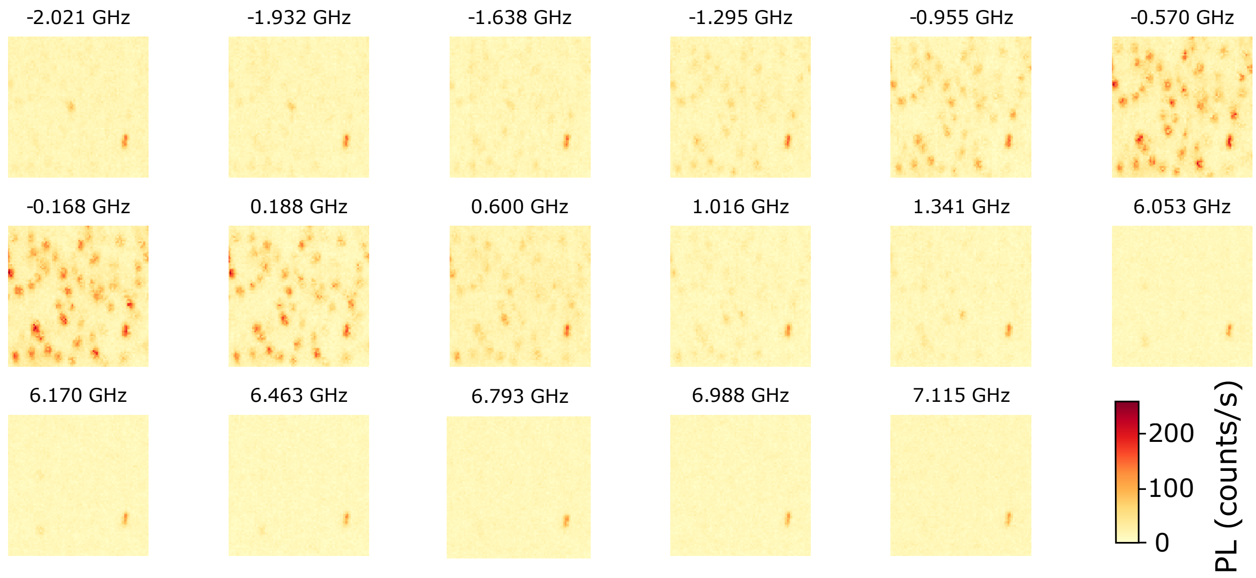

Supplementary Figure 7. **PLE maps (region A3, sample A).** Photoluminescence maps for different detunings of the telecom excitation laser for the isotopically-enriched sample. Each map shows a 12 µm by 12 µm area. For these measurements, we use an integration time of 1 s per step, an excitation power of 14 µW for the repump laser, and 2.2 µW for the telecom laser.

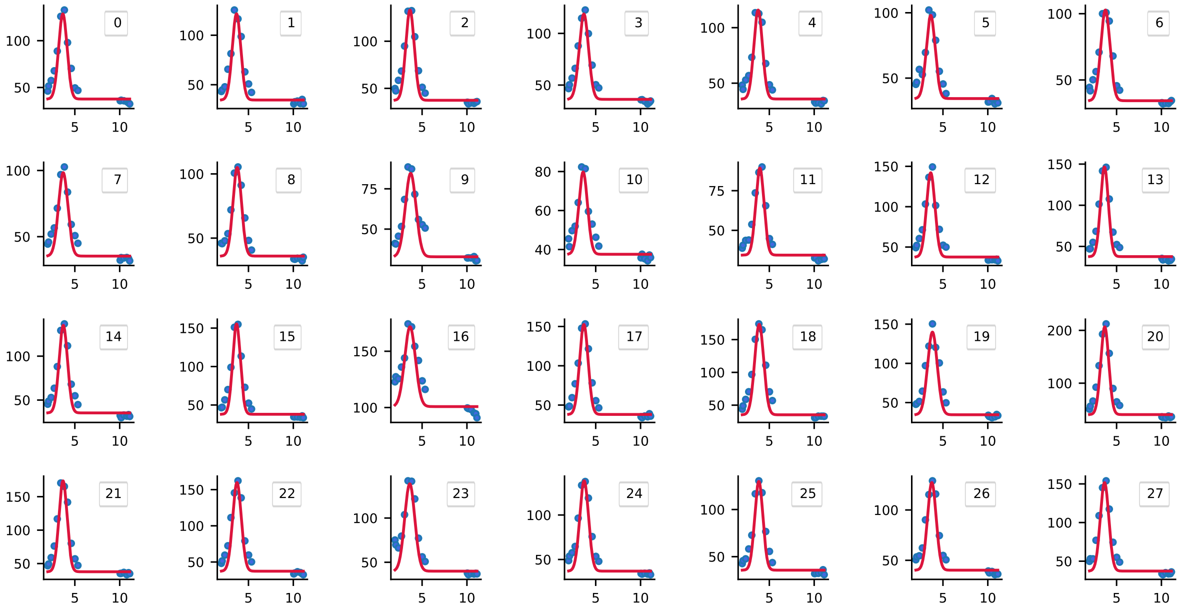

Supplementary Figure 8. **PLE spectra for each spot (region A3, sample A)**. Fitted spectra from 27 PL spots in Supplementary Figure 7. For each sub-plot, the x-axis shows the excitation frequency in GHz, and the y-axis the number of detected counts per second.

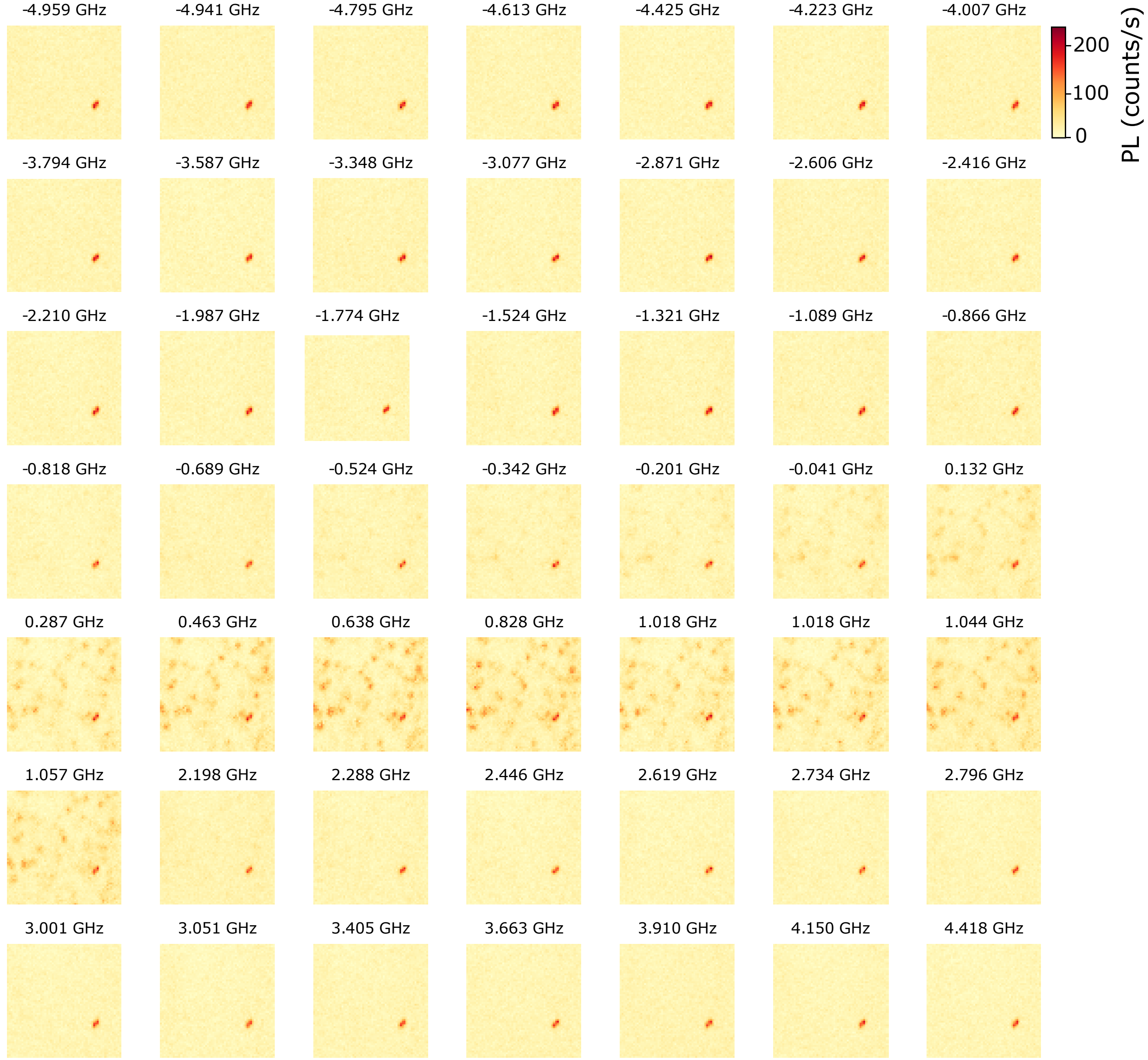

Supplementary Figure 9. **PLE maps (region A4, sample A).** Photoluminescence maps for different detunings of the telecom excitation laser for the isotopically-enriched sample. Each map shows a 12 µm by 12 µm area. For these measurements, we use an integration time of 1 s per step, an excitation power of 14 µW for the repump laser, and 3 µW for the telecom laser.

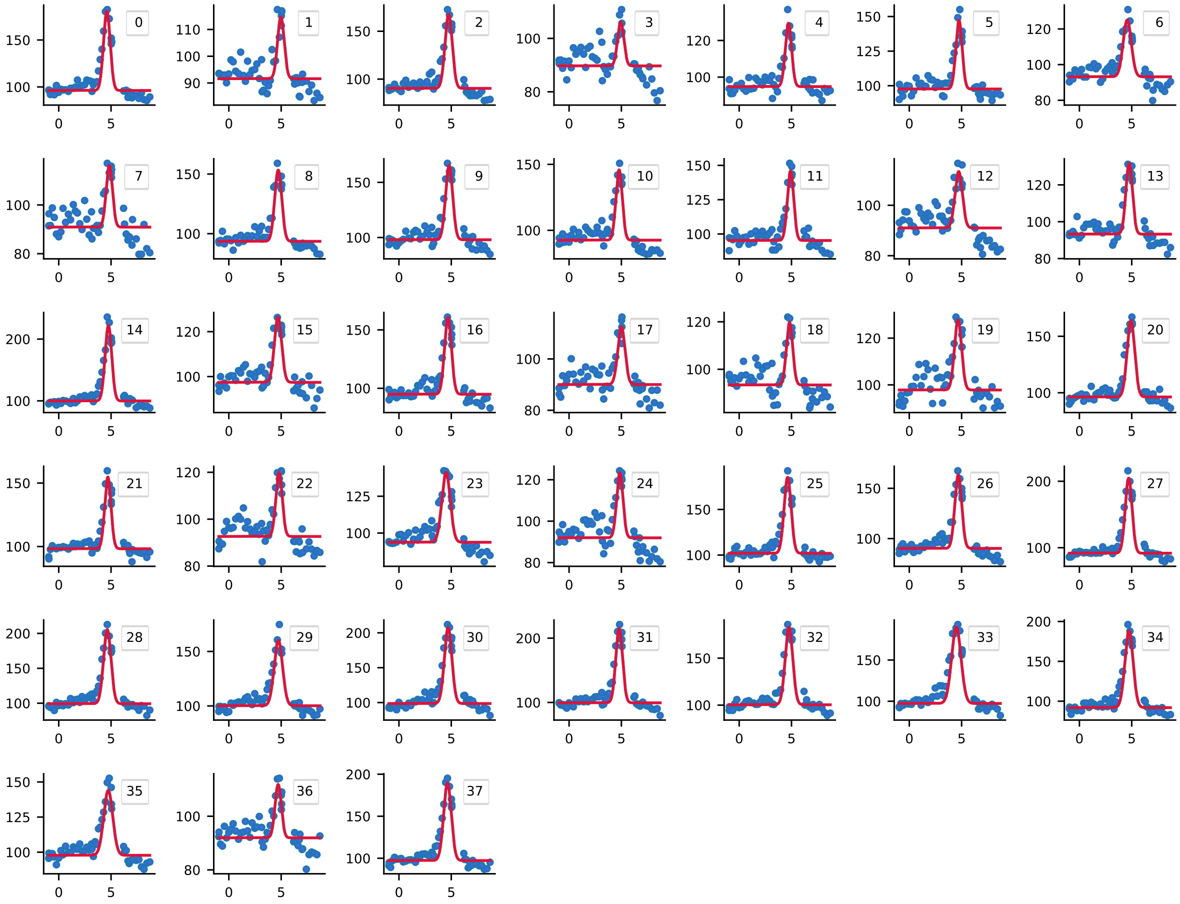

Supplementary Figure 10. **PLE spectra for each spot (region A4, sample A)**. Fitted spectra from 37 PL spots in Supplementary Figure 9. For each sub-plot, the x-axis shows the excitation frequency in GHz, and the y-axis the number of detected counts per second.

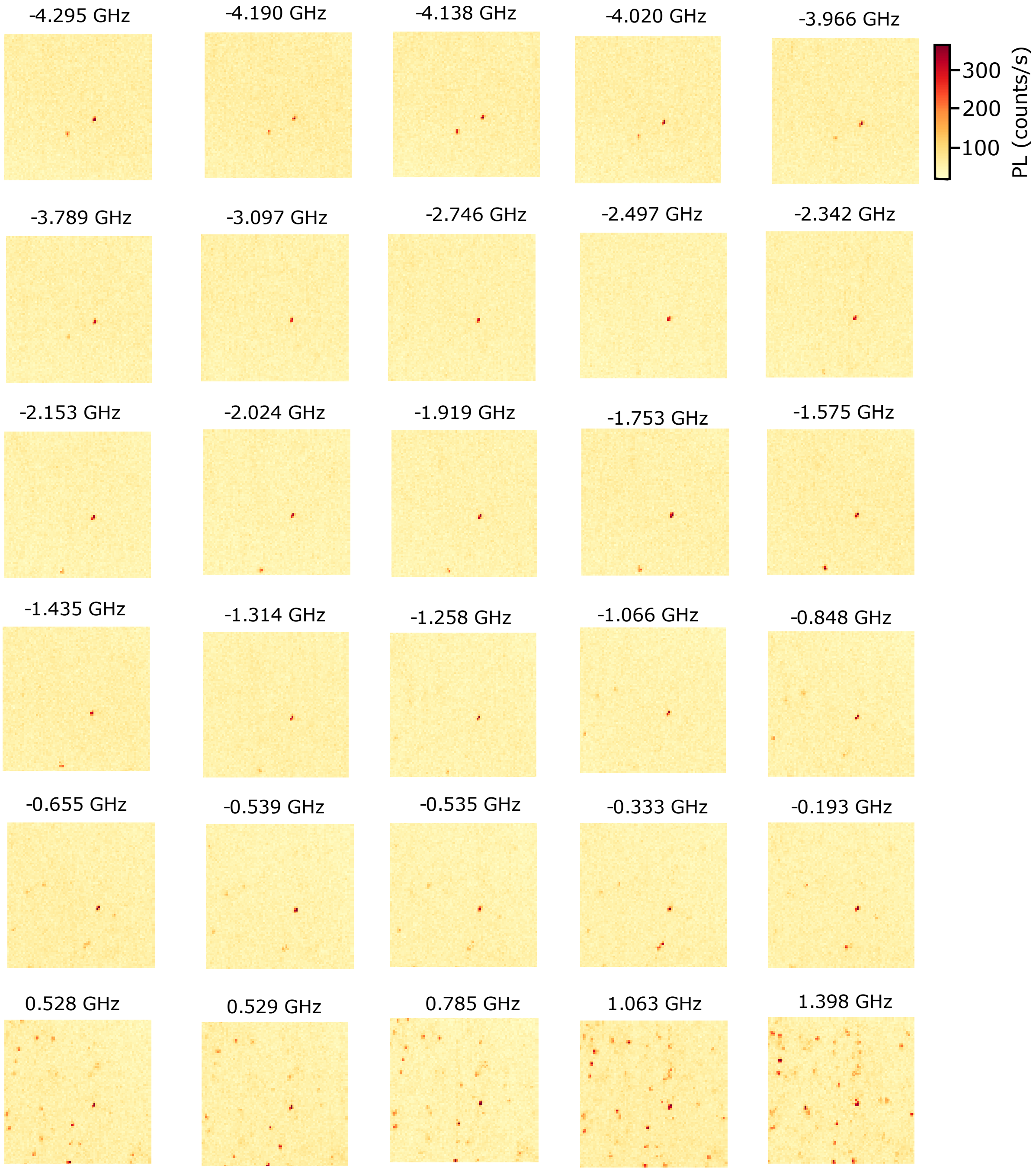

Supplementary Figure 11. **PLE maps, sample B - natural abundance of isotopes (excitation laser frequency range:** -4.3 **to** 1.4 GHz**).** Photoluminescence maps for different detunings of the excitation laser for the sample with natural abundance of Si and C isotopes. Each map shows a 30 µm by 30 µm area. A PL spot is visible around the centre of all maps: its PLE spectrum is very broad and not associated to a V centre. For these measurements, we use an integration time of 1 s per step, an excitation power of 14 µW for the repump laser, and 4 µW for the telecom laser.

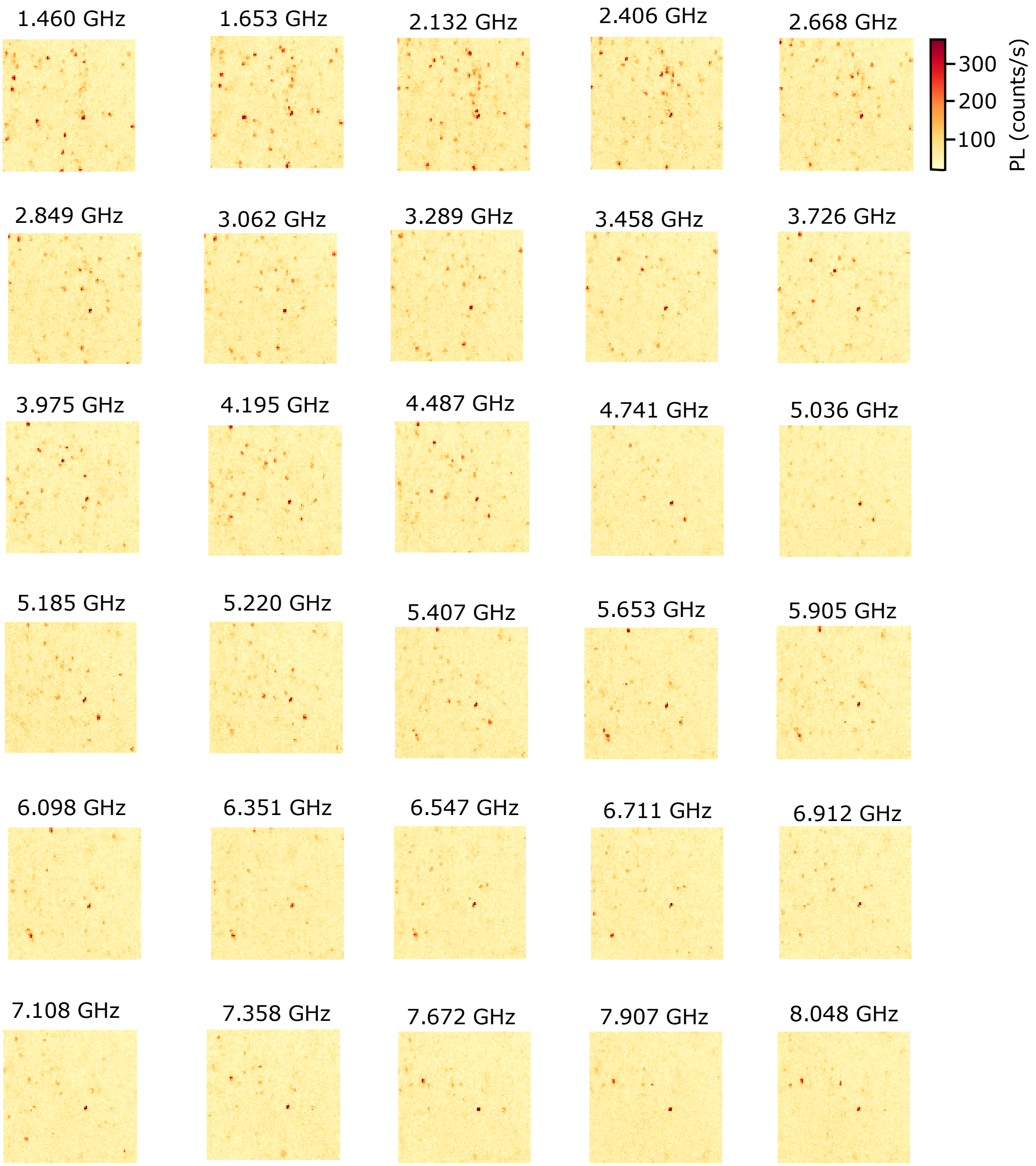

Supplementary Figure 12. **PLE maps, sample B - natural abundance of isotopes (excitation laser frequency range:** 1.4 **to** 8 GHz**).** Photoluminescence maps for different detunings of the excitation laser for the sample with natural abundance of Si and C isotopes. Each map shows a 30 µm by 30 µm area. For these measurements, we use an integration time of 1 s per step, an excitation power of 14 µW for the repump laser, and 4 µW for the telecom laser.

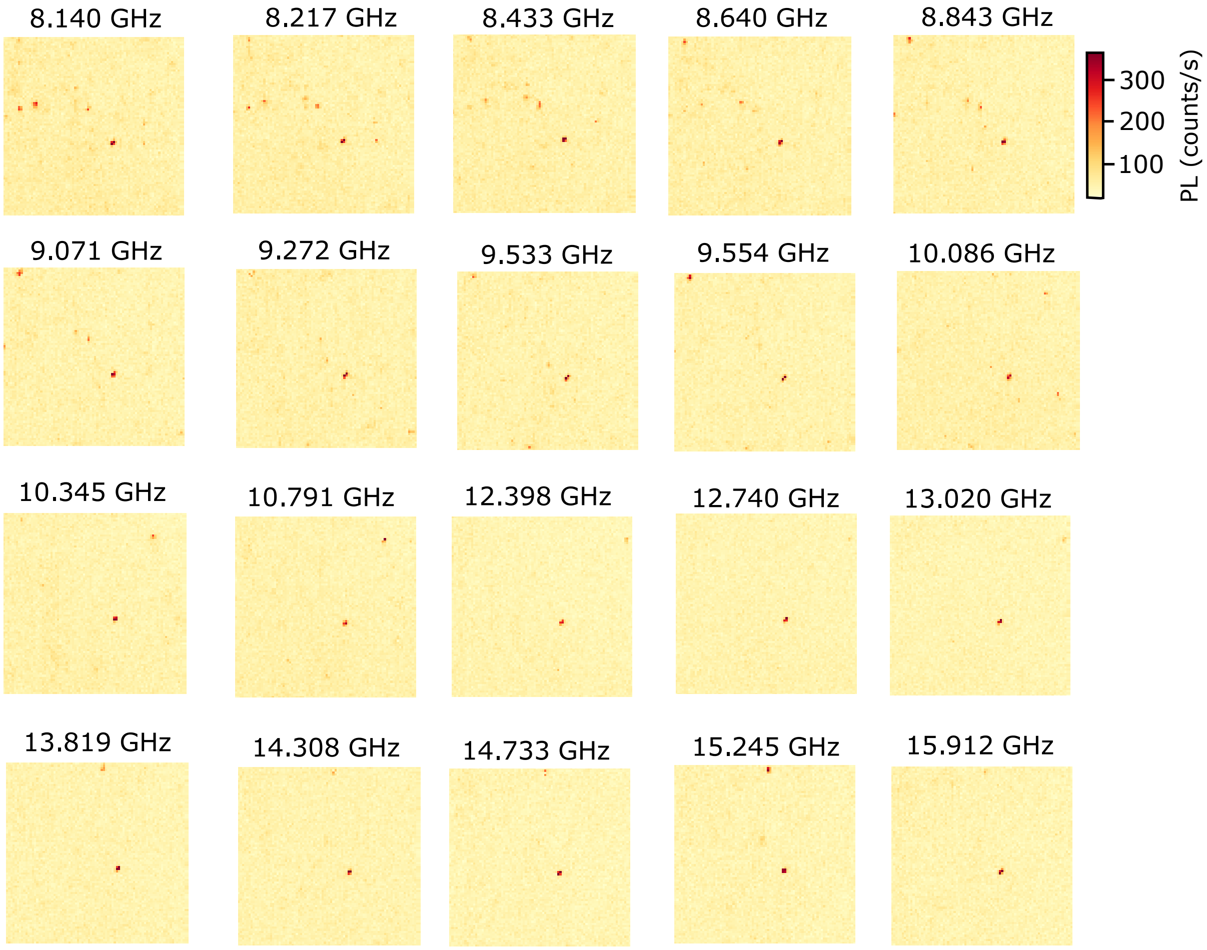

Supplementary Figure 13. **PLE maps, sample B - natural abundance of isotopes (excitation laser frequency range: 8 to 16 GHz).** Photoluminescence maps for different detunings of the excitation laser for the sample with natural abundance of Si and C isotopes. Each map shows a 30 µm by 30 µm area. A PL spot is visible around the centre of all maps: its PLE spectrum is very broad and not associated to a V centre. For these measurements, we use an integration time of 1 s per step, an excitation power of 14 µW for the repump laser, and 4 µW for the telecom laser.

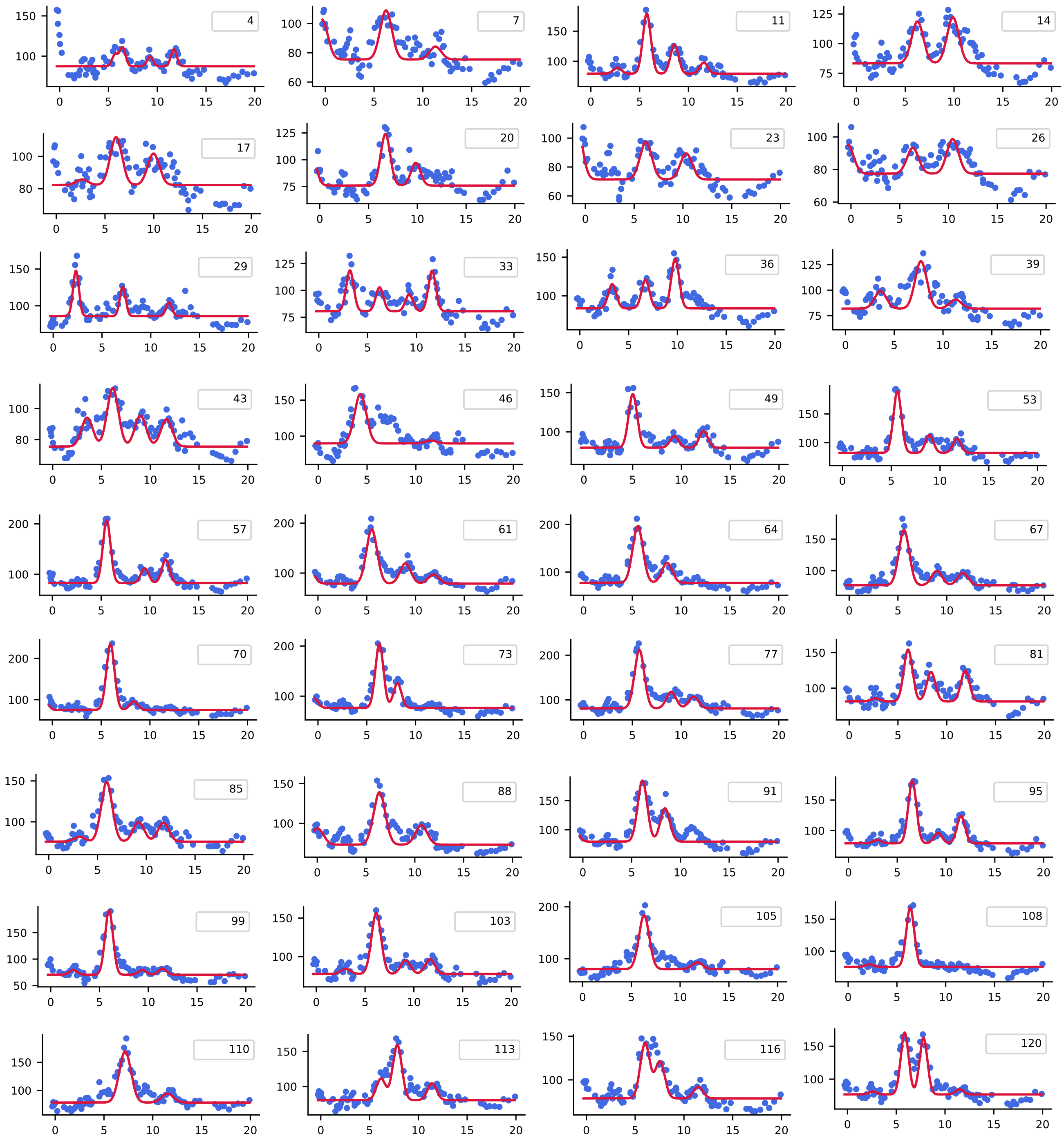

Supplementary Figure 14. **PLE spectra, sample B: natural abundance of isotopes (part I)**. Fitted spectra from 36 PL spots in Supplementary Figure 12. For each sub-plot, the x-axis shows the excitation frequency in GHz, and the y-axis the number of detected counts per second.

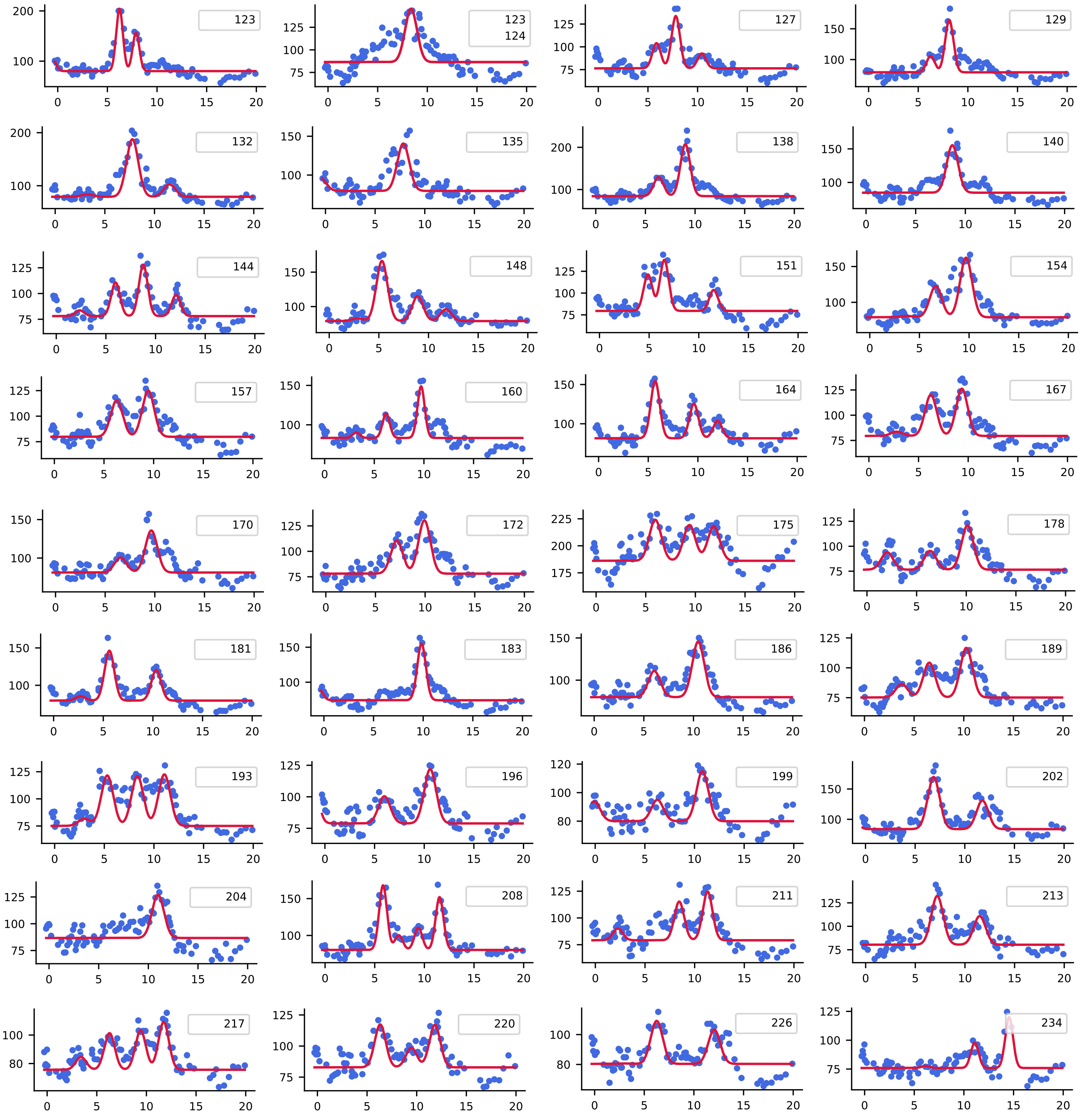

Supplementary Figure 15. **PLE spectra, sample B: natural abundance of isotopes (part II)**. Fitted spectra from 36 PL spots in Supplementary Figure 12. PLE spectra for 36 more spots are reported in Extended Data 4. For each sub-plot, the x-axis shows the excitation frequency in GHz, and the y-axis the number of detected counts per second.

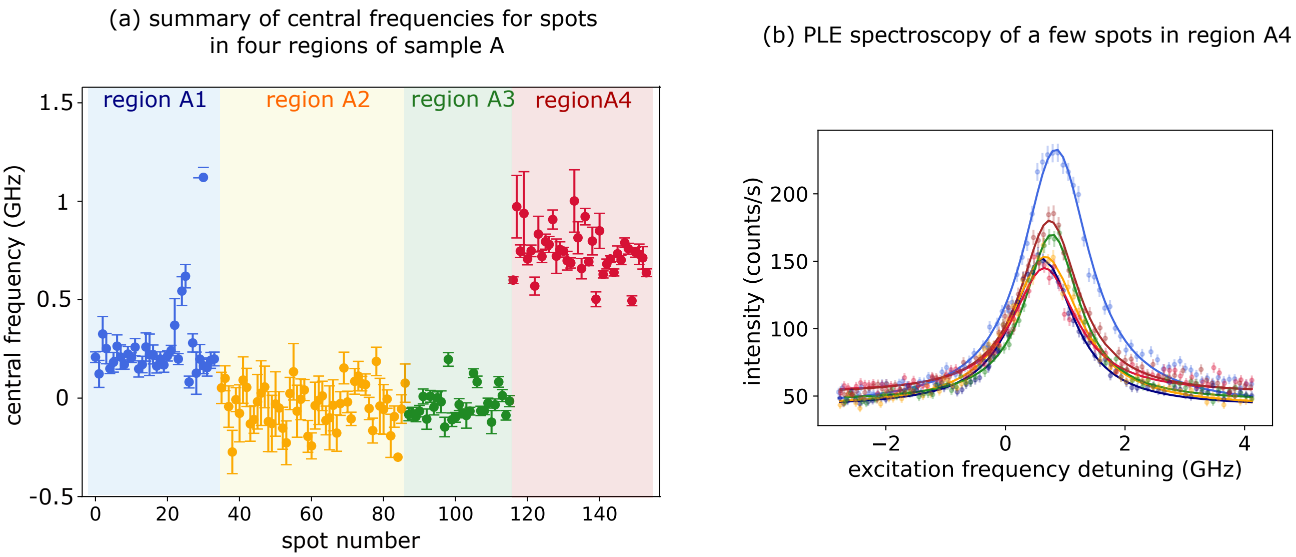

Supplementary Figure 16. **Inhomogeneous distribution of the vanadium emitters in four regions of the sample.** (a) Summary of central frequencies for 150 vanadium centres in four different regions of the sample. In each region, the inhomogeneous distribution features a standard deviation of about 100 MHz. Data for region A4 were collected after the cryostat was warmed up to room temperature and subsequently cooled down to 4 K. The error bars are extracted from the fits of the peaks (i.e., PL spots in the maps). (b) PLE spectroscopy of 6 spots in region A4, to confirm the narrow inhomogeneous distribution observed in the sequence of frequency-resolved PLE maps. The error bars correspond to the Poisson noise of the photon counts.

[1] G. Wolfowicz, C. P. Anderson, B. Diler, O. G. Poluektov, F. J. Heremans, and D. D. Awschalom, Science Advances **6**, eaaz1192 (2020).

[2] N. Achtziger and W. Witthuhn, Applied Physics Letters **71**, 110 (1997).

[3] N. Achtziger and W. Witthuhn, Physical Review B **57**, 12181 (1998).

[4] A. O. Evwaraye, S. R. Smith, and W. C. Mitchel, Journal of Applied Physics **79**, 253 (1996).

[5] J. Baur, M. Kunzer, and J. Schneider, physica status solidi (a) **162**, 153 (1997).

[6] W. C. Mitchel, R. Perrin, J. Goldstein, A. Saxler, M. Roth, S. R. Smith, J. S. Solomon, and A. O. Evwaraye, Journal of Applied Physics **86**, 5040 (1999).

[7] J. M. Langer and H. Heinrich, Phys. Rev. Lett. **55**, 1414 (1985).

[8] W. J. Choyke, Mat. Res. Bull. **4**, S141 (1969).

[9] B. Tissot and G. Burkard, Physical Review B **104**, 064102 (2021).

[10] T. Astner, P. Koller, C. M. Gilardoni, J. Hendriks, N. Son, I. Ivanov, J. Hassan, C. van der Wal, and M. Trupke, (2022), arXiv:2206.06240.

[11] B. Tissot and G. Burkard, Physical Review B **103**, 064106 (2021).

[12] B. Tissot, M. Trupke, P. Koller, T. Astner, and G. Burkard, Phys. Rev. Research **4**, 033107 (2022).

[13] L. Bergeron, C. Chartrand, A. T. K. Kurkjian, K. J. Morse, H. Riemann, N. V. Abrosimov, P. Becker, H.-J. Pohl, M. L. W. Thewalt, and S. Simmons, PRX Quantum **1**, 020301 (2020).

[14] E. R. MacQuarrie, C. Chartrand, D. B. Higginbottom, K. J. Morse, V. A. Karasyuk, S. Roorda, and S. Simmons, New Journal of Physics **23**, 103008 (2021).

[15] S. B. van Dam, M. Walsh, M. J. Degen, E. Bersin, S. L. Mouradian, A. Galiullin, M. Ruf, M. IJspeert, T. H. Taminiau, R. Hanson, and D. R. Englund, Physical Review B **99**, 161203 (2019).

[16] L. J. Rogers, K. D. Jahnke, T. Teraji, L. Marseglia, C. Müller, B. Naydenov, H. Schauffert, C. Kranz, J. Isoya, L. P. McGuinness, and F. Jelezko, Nature Communications **5**, 4739 (2014).

[17] R. E. Evans, A. Sipahigil, D. D. Sukachev, A. S. Zibrov, and M. D. Lukin, Phys. Rev. Applied **5**, 044010 (2016).

[18] R. Nagy, D. B. R. Dasari, C. Babin, D. Liu, V. Vorobyov, M. Niethammer, M. Widmann, T. Linkewitz, I. Gediz, R. Stöhr, H. B. Weber, T. Ohshima, M. Ghezellou, N. T. Son, J. Ul-Hassan, F. Kaiser, and J. Wrachtrup, Applied Physics Letters **118**, 144003 (2021).

[19] A. Gritsch, L. Weiss, J. Früh, S. Rinner, and A. Reiserer, Phys. Rev. X **12**, 041009 (2022).

[20] A. M. Dibos, M. Raha, C. M. Phenicie, and J. D. Thompson, Phys. Rev. Lett. **120**, 243601 (2018).

[21] S. Chen, M. Raha, C. M. Phenicie, S. Ourari, and J. D. Thompson, Science **370**, 592 (2020).

[22] W. Redjem, A. Durand, T. Herzig, A. Benali, S. Pezzagna, J. Meijer, A. Kuznetsov, H. S. Nguyen, S. Cueff, J.-M. Gerard, I. Robert-Philip, B. Gil, D. Caliste, P. Pochet, M. Abbarchi, V. Jacques, A. Dreau, and G. Cassabois, Nature Electronics **3**, 1 (2020).

[23] A. Durand, Y. Baron, W. Redjem, T. Herzig, A. Benali, S. Pezzagna, J. Meijer, A. Y. Kuznetsov, J.-M. Gérard, I. Robert-Philip, M. Abbarchi, V. Jacques, G. Cassabois, and A. Dréau, Phys. Rev. Lett. **126**, 083602 (2021).

[24] C. Chartrand, L. Bergeron, K. J. Morse, H. Riemann, N. V. Abrosimov, P. Becker, H.-J. Pohl, S. Simmons, and M. L. W. Thewalt, Phys. Rev. B **98**, 195201 (2018).

[25] A. Tiranov, A. Ortu, S. Welinski, A. Ferrier, P. Goldner, N. Gisin, and M. Afzelius, Phys. Rev. B **98**, 195110 (2018).

[26] R. Ahlefeldt, N. Manson, and M. Sellars, Journal of Luminescence **133**, 152 (2013).

[27] R. L. Ahlefeldt, M. R. Hush, and M. J. Sellars, Phys. Rev. Lett. **117**, 250504 (2016).

[28] D. Serrano, S. K. Kuppusamy, B. Heinrich, O. Fuhr, D. Hunger, M. Ruben, and P. Goldner, Nature **603**, 241 (2022).
